# Computational identification of *Azadirachta indica* compounds targeting trypanothione reductase in *Leishmania infantum*

**DOI:** 10.1093/bioadv/vbaf318

**Published:** 2025-12-17

**Authors:** Onile Olugbenga Samson, Olukunle Samuel, Fadahunsi Adeyinka Ignatius, Onile Tolulope Adelonpe, Momoh Abdul, Kolawole Oladipo, Afolabi Titilope Esther, Raji Omotara, Hassan Nour, Samir Chtita

**Affiliations:** Department of Biological Sciences, Biotechnology Programme, Elizade University, Ilara-Mokin, Ondo State 340001, Nigeria; Department of Biological Sciences, Biotechnology Programme, Elizade University, Ilara-Mokin, Ondo State 340001, Nigeria; Department of Biological Sciences, Biotechnology Programme, Elizade University, Ilara-Mokin, Ondo State 340001, Nigeria; Department of Biological Sciences, Microbiology Programme, Elizade University, Ilara-Mokin, Ondo State 340001, Nigeria; Department of Biological Sciences, Microbiology Programme, Elizade University, Ilara-Mokin, Ondo State 340001, Nigeria; Department of Microbiology, Laboratory of Molecular Biology, Immunology and Bioinformatics, Adeleke University, Ede, Osun State 232104, Nigeria; Department of Molecular Biology and Biotechnology, Chrisland University, Abeokuta, Ogun State 230001, Nigeria; Department of Biological Sciences, Biotechnology Programme, Elizade University, Ilara-Mokin, Ondo State 340001, Nigeria; Laboratory of Analytical and Molecular Chemistry, Faculty of Sciences Ben M’Sik, Hassan II University of Casablanca, Casablanca 7955, Morocco; Laboratory of Analytical and Molecular Chemistry, Faculty of Sciences Ben M’Sik, Hassan II University of Casablanca, Casablanca 7955, Morocco

## Abstract

**Motivation:** *Leishmania infantum* is the primary cause of VL, and its trypanothione reductase (TR) creates a favorable environment in the host, making TR an attractive drug target. This study aims to identify potential TR inhibitors from *Azadirachta indica* phytochemicals using molecular modeling techniques.

**Results:** Sixty compounds from *A. indica* were screened via molecular docking for their binding affinity to TR, followed by binding free energy calculations. Drug-likeness, pharmacokinetics, and toxicity properties of the hit compounds were then evaluated. The top compounds were subjected to a 100 ns molecular dynamics (MDs) simulation to further assess the stability of their interaction with TR. Ten of the screened compounds exhibited higher affinity for TR compared to miltefosine (standard drug), with docking scores ranging from −3.501 to −8.482 kcal/mol, compared to miltefosine’s −3.231 kcal/mol. All the drug-like hit compounds showed favorable pharmacokinetics and toxicity profiles and their binding free energies indicated stable interactions. MDs simulations confirmed that these interactions persisted for most of the simulation time, confirming the stability and potential efficacy of the compounds as TR inhibitors.

**Availability and Implementation:** This study identifies isorhamnetin, meliantriol, and quercetin as promising candidates for further *in vitro* and *in vivo* evaluation for the development of TR inhibitors against *L. infantum*.

## 1 Introduction

*Leishmania*sis, a parasitic disease transmitted by vectors, exhibits high morbidity and a wide range of clinical manifestations (Onile *et al.* 2022). The three primary forms of leishmaniasis are visceral leishmaniasis (VL), cutaneous leishmaniasis (CL), and mucocutaneous leishmaniasis (MCL) (Sasidharan and Saudagar 2021), of which VL is the most lethal form, with nearly 90% of global VL cases occurring in seven countries: Brazil, Ethiopia, India, Kenya, Somalia, South Sudan, and Sudan. Annually, there occur 500 000 new VL cases and 50 000 associated fatalities (Scarpini *et al.* 2022), with *Leishmania infantum* and *Leishmania donovani* being the primary etiological organisms. In the tropical and sub-tropical regions of the world, *L. infantum* is the predominant etiological organism, and it creates a favorable biological milieu in the human host through the complex action of its trypanothione reductase (TR) (Bourhia *et al.* 2024b). TR of *L. infantum* is crucial for maintaining trypanothione in its reduced state, which is essential for cellular redox balance in trypanosomatid parasites, including *Trypanosoma* and *Leishmania* species (Field *et al.* 2017). Notably, TR mediates the reduction of trypanothione disulfide (TS2) back to its dithiol form, trypanothione (TSH2), thereby maintaining redox homeostasis and protecting the parasite from oxidative stress by neutralizing reactive oxygen species (ROS) (Battista *et al.* 2020). Over the years, TR has been identified as a suitable drug target for combating leishmaniasis caused by *L. infantum* due to reasons including its vital role in parasite survival and absence in the host, where glutathione reductase serves a similar function. Despite their similarities, TR and GR have distinct thiol-binding sites, allowing for the potential development of TR-specific drugs that do not affect the function of GR in the human host (Inacio *et al.* 2021). Chemotherapy is the primary treatment for VL. Current available medications include injectable paromomycin, oral miltefosine, sodium stibogluconate, meglumine antimoniate, pentavalent antimonial compounds, and two formulations of amphotericin B: lipid formulations and free deoxycholate, the latter of which is no longer used (Alves *et al.* 2018). Miltefosine, an alkyl phospholipid initially developed as an antineoplastic agent for breast cancer, is the only oral drug available for VL treatment, it functions by inducing the macrophages to produce nitric oxide, which triggers a series of reactions that leads to the disruption the parasite’s plasma membrane and mitochondrial structure, and ultimately death (Mazire *et al.* 2022). Despite its efficacy, MF is contraindicated during pregnancy due to its teratogenic potential, and its main side effects include dehydration, vomiting, and diarrhea (Kumari *et al.* 2021). Consequently, the process of identifying safer alternatives with lesser side effects that can be used in place of miltefosine is ongoing, and medicinal plants are a viable source for the identification of antiparasitic compounds (Bourhia *et al.* 2024b). *Azadirachta indica*, commonly known as neem, is a widely used medicinal tree found in tropical regions worldwide and is renowned for its pharmacological properties, which include anti-inflammatory, anti-hyperglycaemic, anti-ulcer, anticancer, antioxidant, and antidiabetic properties (Agrawal *et al.* 2020, Islas *et al.* 2020). Notably, all parts of the neem tree, including the bark, leaves, sap, fruit, seeds, and twigs, exhibit a broad range of pharmacological effects, and its bark extract has been shown to inhibit HSV-1 and murine-β-Coronavirus replication, spread, and fusion *in vitro* (Sarkar *et al.* 2022). Also, the aqueous extracts of the leaves have been reported to exhibit parasiticidal effects on *Zeylanicobdella arugamensis* and have been reported to be a good source of metabolites with antiparasitic potential (Venmathi Maran *et al.* 2021). Hence, this study aims to virtually screen compounds from the bark and leaves of *A. indica* for the identification of compounds with the potential to inhibit TR of *L. infantum* using computer-aided drug discovery approaches, including molecular docking, drug-likeness profiling, pharmacokinetics screening, binding-free energy calculation, and MDs simulation (Frye *et al.* 2021, Omoboyede *et al.* 2023).

## 2 Methods

### 2.1 Retrieval and optimization of ligand structures

The phytochemicals present in *A. indica* were identified from the Indian Medicinal Plants, Phytochemistry and Therapeutics curated database (imsc.res.in) (Mohanraj *et al.* 2018). Their chemical structures were retrieved in three-dimensional (3D) structure data format (SDF). After the structure was retrieved, they were subsequently imported into the Maestro interface and subjected to a structural optimization pipeline using the LigPrep module (Ogbodo *et al.* 2023).

### 2.2 Target structure retrieval and preparation

The 3D structure of TR in complex with a compound “4a,” determined by X-ray diffraction with a resolution of 2.50 Å, was retrieved from the Protein Data Bank (PDB) (https://www.rcsb.org/) using the PDB ID: 6T95 (De Lucio *et al.* 2022). This structure offers precise atomic coordinates, ensuring reliable ligand binding predictions. Noteworthy, other available structures had higher resolution values, which generally correspond to lower-quality models with less precise atomic positioning. The protein structure was integrated into the Schrodinger Maestro software environment and was prepared using the protein preparation workflow. This preparation procedure involved several critical steps, including the addition of hydrogens, removal of water molecules, het state generation at pH of 7.0 ± 2.0, and PROPKA-based structural optimization (Ogbodo *et al.* 2023).

### 2.3 Receptor grid generation

To identify the amino acid residues constituting the active site TR, the receptor grid generation tool in Schrödinger Maestro was utilized. This tool generates a 3D grid representing the spatial arrangement of amino acid residues in the active site based on the cognate ligand’s position for accurate and targeted docking simulations (Iwaloye *et al.* 2020). Notably, this structure has been previously used to identify 1,2,3-triazolium salt-based inhibitors with enhanced antileishmanial potency and selectivity; hence, the relevance of the identified active site for inhibitor design (De Lucio *et al.* 2022).

### 2.4 Molecular docking simulation

To investigate ligand interactions and affinities with the amino acid residues of TR, molecular docking simulations were performed using the Glide module of Schrödinger Maestro (Release 2024-1). The docking grid was centered on the co-crystallized ligand in the X-ray structure of PDB ID: 6T95 to define the biologically relevant binding site. Subsequently, docking was conducted in two stages: first, the Standard Precision (SP) algorithm, which provides rapid evaluation of ligand binding poses and preliminary scoring, was applied to screen the full compound library. Subsequently, the Extra Precision (XP) algorithm, which uses a more rigorous scoring function that penalizes unfavorable interactions and improves discrimination between high- and low-affinity ligands, was applied to the top 10 compounds from the SP results.

### 2.5 Binding free energy calculation

After the molecular docking simulation, the hit compounds’ interactions with the protein were assessed for stability using Maestro’s Molecular Mechanics with Generalized Born and Surface Area (MM-GBSA) post-docking methodology (Ogbodo *et al.* 2023). During this process, the Variable Solvent Generalized Born (VSGB) solvation model and the OPLS3 force field were employed with other parameters kept default, and the hit compounds’ binding free energies (*G*_bind_) were calculated using the equations below (Tripathi *et al.* 2013):


$$\Delta G_{\text{bind}}=\Delta E+\Delta G_{\text{solv}}+\Delta \text{GSA}$$

$$\Delta E=E_{\text{complex}}-E_{\text{protein}}-E_{\text{ligand}}$$

The energy change (Δ*E*) is determined by the difference between the minimized energies of the protein–inhibitor complex (*E*_complex_), protein (*E*_protein_), and inhibitor (*E*_ligand_).


$$\Delta G_{\text{solv}}=G_{\text{solv}(\text{complex})}-G_{\text{solv}(\text{protein})}-G_{\text{solv}(\text{ligand})}$$

The solvation-free energy change (Δ*G*_solv_) accounts for the difference in solvation energies between the complex (G*G*_solv(complex)_), protein (*G*_solv(protein)_), and inhibitor (*G*_solv(ligand)_).


$$\Delta \text{GSA}={\text{GSA}}_{\left(\text{complex}\right)}-{\text{GSA}}_{\left(\text{protein}\right)}-{\text{GSA}}_{\left(\text{ligand}\right)}$$

(Lyne *et al.* 2006).

The surface area energy change (ΔGSA) reflects the difference in surface area energies between the complex (GSA_(complex)_), protein (GSA_(protein)_), and inhibitor (GSA_(ligand)_).

### 2.6 Drug-likeness and pharmacokinetics property evaluation

The adherence of the hit compounds to Lipinski’s rule of five (Ro5) was assessed Maestro’s QikProp module (Lipinski *et al.* 2001). Subsequently, the absorption, distribution, metabolism, excretion, and toxicity (ADMET) profiles of the druglike hit compounds were assessed using the pkCSM server (Pires *et al.* 2015). The SMILES of the compounds (Supplementary Table 1), and results were interpreted according to the thresholds described in the original references.

### 2.7 MDs simulation

The behavior of the complexes under investigation was dynamically studied in an aqueous environment through the implementation of MD simulations. The MD simulations were carried out using Maestro-Schrödinger v2021-3. All the simulations were performed by applying the OPLS3e force field (Roos *et al.* 2019). The systems being examined were immersed in water using the SPC solvent model, with the addition of sodium and chloride ions to maintain neutrality. To confine the simulation space, the systems were enclosed within an orthorhombic box. Subsequently, they underwent NVT equilibration at 300 K for 1 nanosecond, followed by NPT equilibration at 1 bar for an additional nanosecond (ns). Finally, the equilibrated systems were subjected to a 100 ns simulation to study their MDs.

## 3 Results

### 3.1 *A. indica* compounds exhibited high affinities for TR

**Figure 1.**
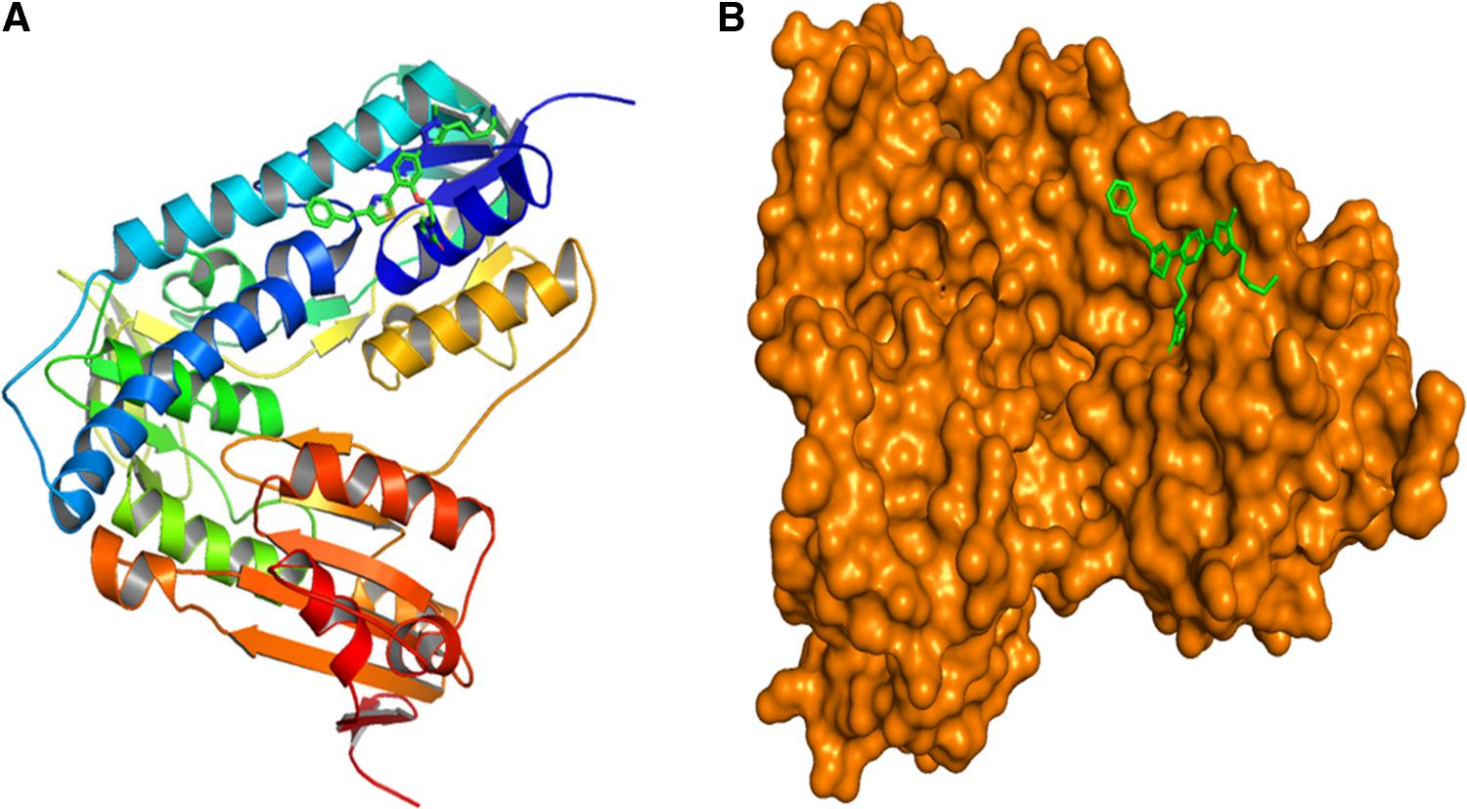
Surface and cartoon representations of the modeled Trypanothione reductase structure showing the binding pocket and co-crystallized ligand. (A) Cartoon representation of modeled Trypanothione reductase structure showing the binding pocket and co-crystallized ligand. (B) Surface representation of Trypanothione reductase shows the enzyme complexed with the co-crystallized ligand situated within the defined active site pocket. This view emphazies the interaction of the ligand with surrounding residues consisting the catalytic domain responsible for substrate recognition and redox functionality.

Early in the drug development process, molecular docking is a vital method that enables the virtual screening of a large library of compounds to find promising compounds that interact with certain protein targets linked to diseases. In this study, 60 compounds from the bark and leaves of *A. indica* were docked into the active site of TR, which is depicted in Fig. 1. The docking scores of the hit compounds are presented in Table 1; some of the compounds have higher affinities for TR compared to miltefosine.

**Table 1.** The docking scores of the top 10 compounds and the standard drug (Miltefosine).

S/N	Compound name	Docking score (kcal/mol)
1	Miltefosine	−3.231
2	Rutin	−8.482
3	Nicotiflorin	−8.122
4	Hyperoside	−6.801
5	Quercitrin	−6.362
6	Isorhamnetin	−5.765
7	Meliantriol	−5.621
8	Quercetin	−5.238
9	Nimbinone	−3.974
10	Scopoletin	−3.879
11	Methyl 2,5-dihydroxycinnamate	−3.501

### 3.2 Six hit compounds from *A. indica* possess druglike properties

As presented in Table 2, druglikeness evaluation revealed four of the hit compounds, namely rutin, nicotiflorin, hyperoside, and quercitrin violated the Ro5, as their values for certain parameters exceeded the acceptable threshold of the Ro5. Specifically, the compounds possess hydrogen bond donors and acceptors which exceed the respective 5 and 10 limits stated by the Ro5, hence, they were considered non-druglike.

**Table 2.** The drug-likeness profiles of the hit compounds.

Compound name	mol_MW^a^	donorHB^b^	accptHB^c^	QPlogPo/w^d^	Ro5 violation^e^
Rutin	610.524	9	20.55	−2.398	3
Nicotiflorin	594.525	8	19.8	−1.805	3
Hyperoside	464.382	7	13.75	−1.374	2
Quercitrin	448.382	6	12.05	−0.479	2
Isorhamnetin	316.267	3	5.25	1.229	0
Meliantriol	490.722	4	7.55	4.258	0
Quercetin	302.24	4	5.25	0.405	0
Nimbinone	286.37	1	4.75	2.313	0
Scopoletin	192.171	1	4	0.854	0
Methyl-2,5-dihydroxycinnamate	194.187	2	3.5	1.012	0

a mol_MW is the molecular weight (range: <500).

b donorHB is the number of hydrogen bond donors (range ≤5).

c accptHB is the number of hydrogen bond acceptors (range ≤10).

d QPlogPo/w is the octanol-water partition coefficient (range: −2.0 to 6.5).

e Ro5 violation is the linpisk’s rule of five violation

### 3.3 Six druglike compounds possess desirable ADMET profile

The hit compounds that were deemed to possess druglike properties were subjected to additional examination of their pharmacokinetic properties and the outcomes are presented in Table 3. All the compounds had high absorption potential and were all predicted to have the potential to serve as a substrate of the P-glycoprotein except for scopoletin. They were all predicted to be non-inhibitors of P-glycoprotein I and II and non-substrate of the renal organic cation transporter 2. For their toxicity profiles, none of the compounds was predicted to be AMES toxic and inhibitors of the hERG I and II channels. Also, they predicted to be non-hepatotoxic. Conversely, miltefosine was predicted to be hepatotoxic, inhibit hERG II, and be skin sensitive (Table 3).

**Table 3.** The pharmacokinetics profiles of the hit compound and the standard drug.^a^

Models	Compounds						
	Isorhamnetin	Meliantriol	Quercetin	Nimbinone	Scopoletin	Methyl 2,5-dihydroxycinnamate	Miltefosine
**Absorption**							
HIA	76.014	93.44	77.207	93.587	95.277	75.616	92.021
Water solubility	−3	−5.175	−2.925	−3.233	−2.504	−1.652	−6.149
CaCO_2_ permeability	−0.003	1.045	−0.229	1.308	1.184	0.081	1.049
P-gp (substrate)	Substrate	Substrate	Substrate	Substrate	Non-substrate	Substrate	Non-substrate
P-gp I (inhibitor)	Non-inhibitor	Non-inhibitor	Non-inhibitor	Non-inhibitor	Non-inhibitor	Non-inhibitor	Inhibitor
P-gp II (inhibitor)	Non-inhibitor	Non-inhibitor	Non-inhibitor	Non-inhibitor	Non-inhibitor	Non-inhibitor	Inhibitor
**Distribution**							
Blood-brain barrier	−1.135	−0.512	−0.229	−0.2	−0.299	−0.321	−0.173
Central nervous system permeability	−3.188	−2.073	−0.229	−1.635	−2.32	−2.568	−3.191
Fraction unbound	0.091	0.067	−0.229	0.234	0.363	0.494	0.162
**Metabolism**							
CYP450 2C9 (inhibition)	Non-inhibitor	Non-inhibitor	Non-inhibitor	Non-inhibitor	Non-inhibitor	Non-inhibitor	Non-inhibitor
CYP450 2D6 (substrate)	Non-substrate	Non-substrate	Non-substrate	Non-substrate	Non-substrate	Non-substrate	Non-substrate
CYP450 2D6 (inhibition)	Non-inhibitor	Non-inhibitor	Non-inhibitor	Non-inhibitor	Non-inhibitor	Non-inhibitor	Non-inhibitor
CYP450 3A4 (substrate)	Non-substrate	Substrate	Non-substrate	Non-substrate	Non-substrate	Non-substrate	Substrate
CYP450 3A4 (inhibition)	Non-inhibitor	Non-inhibitor	Non-inhibitor	Non-inhibitor	Non-inhibitor	Non-inhibitor	Non-inhibitor
CYP450 1A2 (inhibition)	Inhibitor	Non-inhibitor	Inhibitor	Inhibitor	Inhibitor	Non-inhibitor	Non-inhibitor
CYP450 2C19 (inhibition)	Non-inhibitor	Non-inhibitor	Non-inhibitor	Inhibitor	Non-inhibitor	Non-inhibitor	Non-inhibitor
**Excretion**							
Renal organic cation transporter 2 (OCT2)	Non-substrate	Non-substrate	Non-substrate	Non-substrate	Non-substrate	Non-substrate	Non-substrate
Total clearance	0.508	0.285	0.407	0.756	0.73	0.726	1.112
**Toxicity**							
AMES toxicity	Non-toxic	Non-toxic	Non-toxic	Non-toxic	Non-toxic	Non-toxic	Non-toxic
Max tolerated dose (human)	0.576	−1.204	0.499	0.614	−0.683	−0.154	0.211
hERG I inhibitor	Non-inhibitor	Non-inhibitor	Non-inhibitor	Non-inhibitor	Non-inhibitor	Non-inhibitor	Non-inhibitor
hERG II inhibitor	Non-inhibitor	Non-inhibitor	Non-inhibitor	Non-inhibitor	Non-inhibitor	Non-inhibitor	Inhibitor
Oral rat acute toxicity	2.407	2.959	2.471	1.95	2.299	2.011	2.655
Hepatotoxicity	Non-toxic	Non-toxic	Non-toxic	Non-toxic	Non-toxic	Non-toxic	Toxic
Skin sensitization	Non-toxic	Non-toxic	Non-toxic	Non-toxic	Non-toxic	Non-toxic	Toxic

a Water solubility is expressed as log mol/L; CaCO_2_ permeability as log Papp in 10^–6^ cm/s; HIA as % absorbed; BBB as log BB; CNS as log PS; Fraction unbound: Fu; Total clearance: log ml/min/kg; Max tolerated dose (human): log mg/kg/day; Oral rat acute toxicity: mol/kg.

### 3.4 Meliantriol’s binding with TR is the most stable

As presented in Table 4, the binding free energies revealed the compounds stably interacted with the amino acid residues that constitute the active site of TR, with meliantriol exhibiting the highest stability with a value of −43.09 kcal/mol. The standard drug (miltefosine) also exhibited a high stability with a value of −37.34 kcal/mol, albeit lower compared to that of meliantriol. Other hit compounds including quercetin and isorhamnetin were also found to be stable, however, scopoletin formed the least stable interaction.

**Table 4.** The binding free energies which depicts the stability of the interaction of the hit compounds and the standard drug (Miltefosine)^a^.

S/N	Compounds	Δ*G*_bind_	Δ*G*_bind_ Coulomb	Δ*G*_bind_ Hbond	Δ*G*_bind_ Lipophilic	Δ*G*_bind_ Packing	Δ*G*_bind_ vdW
1	Isorhamnetin	−36.63	−4.22	−1.63	−10.86	−4.06	−31.38
2	Meliantriol	−43.09	−12.26	−2.57	−10.86	0	−32.99
3	Quercetin	−32.81	−20.6	−2.95	−6.88	−1.82	−21.61
4	Nimbinone	−34.22	−8.53	−0.49	−13.8	−0.03	−26.81
5	Scopoletin	−29.81	−2.42	−1.3	−11.44	−2.85	−16.78
6	Methyl 2,5-dihydroxycinnamate	−35.31	−17.93	−1.1	−11.13	−1.38	−15.98
7	Miltefosine	−37.34	53.25	−0.65	−19.77	0	−35.72

a All energy contributions (Δ*G*_bind_, Δ*G*_bind_ Coulomb, Δ*G*_bind_ Hbond, Δ*G*_bind_ Lipophilic, Δ*G*_bind_ Packing, Δ*G*_bind_ vdW) are expressed in kcal/mol.

### 3.5 The hit compounds interacted with core amino acid residues in TR’s active site

**Figure 2.**
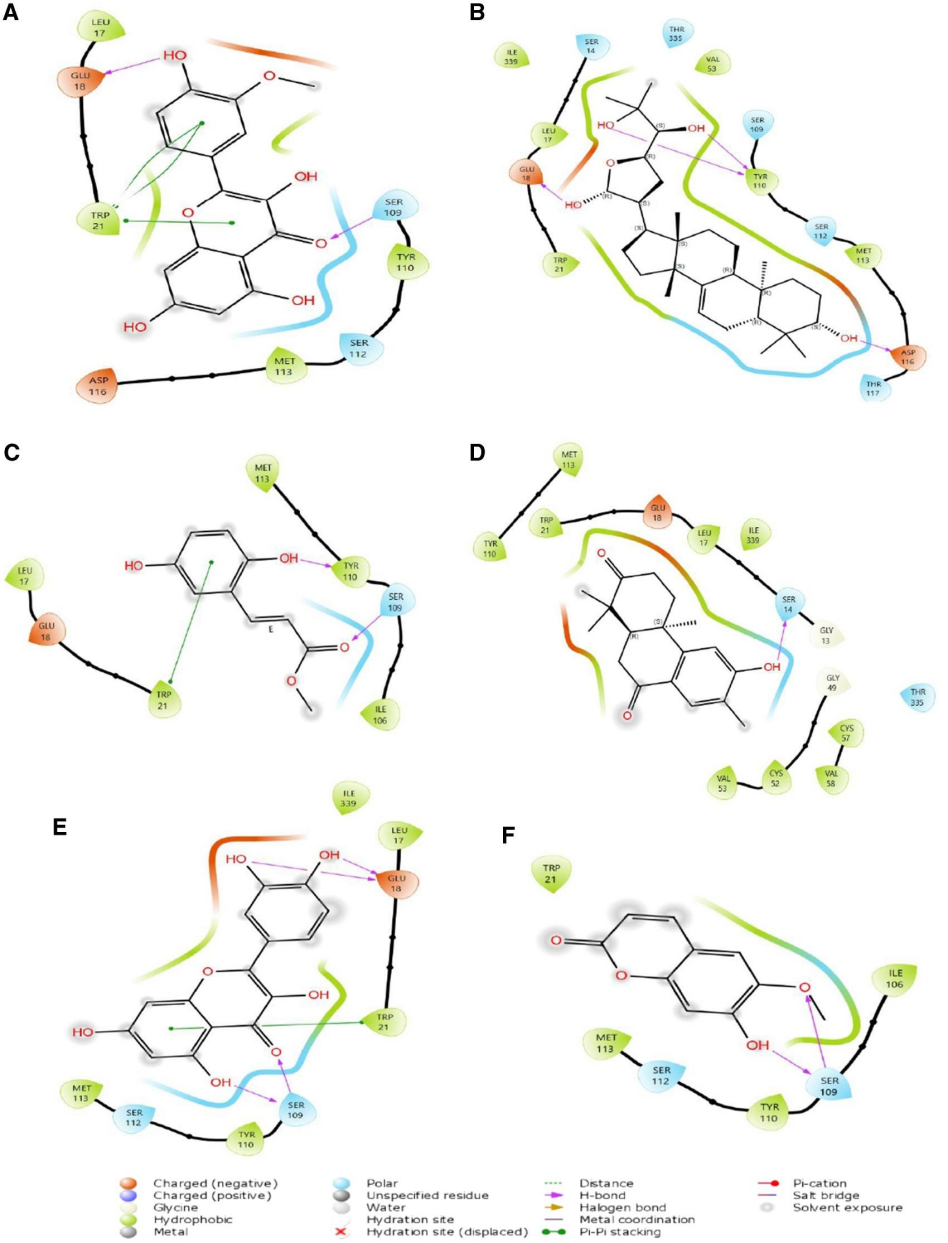
Two-dimensional interaction diagrams of the top hit compounds bound to TryR. The diagrams show the amino acid residues involved in the interactions and the types of bonds formed within the binding pocket. The color codes represent the nature of the interactions, as indicated in the legend. (A) TryR–Isorhamnetin; (B) TryR–Meliantriol; (C) TryR–Methyl 2,5-dihydroxycinnamate; (D) TryR–Nimbinone; (E) TryR–Quercetin; (F) TryR–Scopoletin.

As evident in Fig. 2, the hit compounds formed diverse interactions with critical amino acid residues in the active site of TR, with bonds including hydrogen bond, pi-pi stacking, positively and negatively charged interactions, and polar interactions.

### 3.6 The complexes showed stability during simulation period

**Figure 3.**
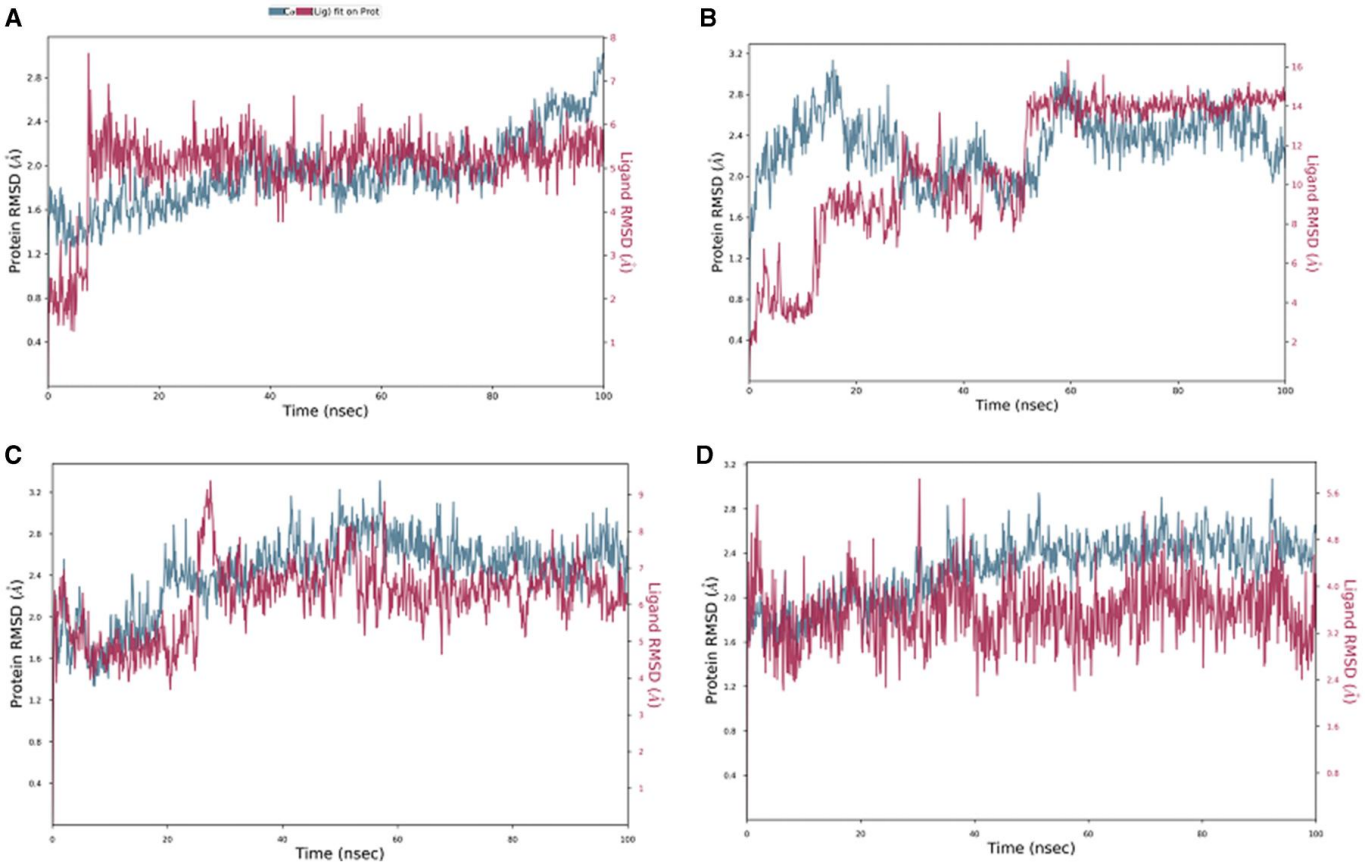
RMSD profiles of TryR and its ligand complexes over a 100 ns molecular dynamics (MD) simulation. The protein backbone RMSD (blue) and ligand RMSD (pink) were monitored to assess the structural stability of each complex. (A) TryR–Isorhamnetin, (B) TryR–Meliantriol, (C) TryR–Miltefosine, and (D) TryR–Quercetin.

**Figure 4.**
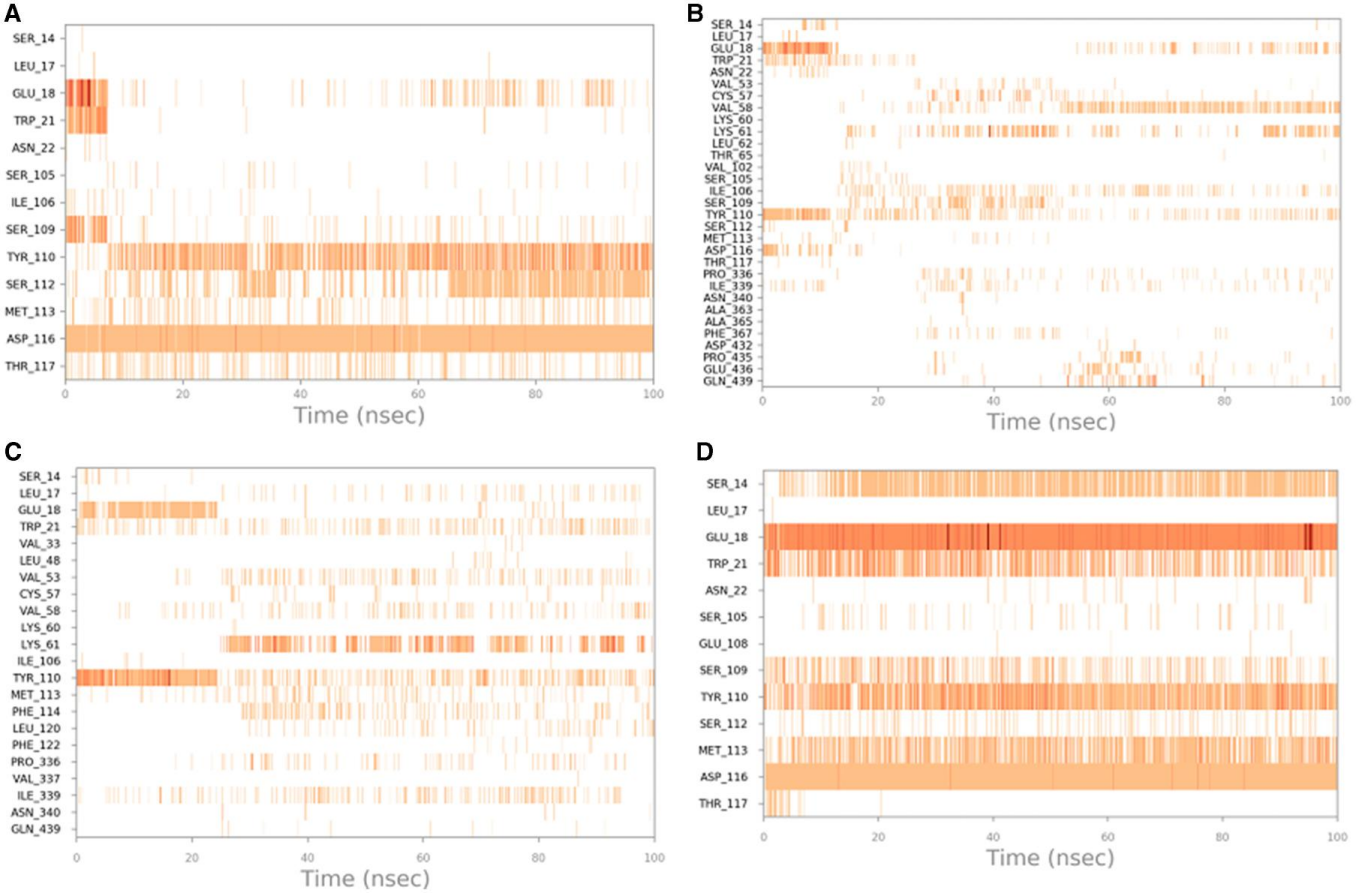
Timeline representation of the ligand-protein interactions: (A) TryR-Isorhamnetin; (B) TryR- Meliantriol; (C) TryR-miltefosine; (D) TryR-Quercetin.

**Figure 5.**
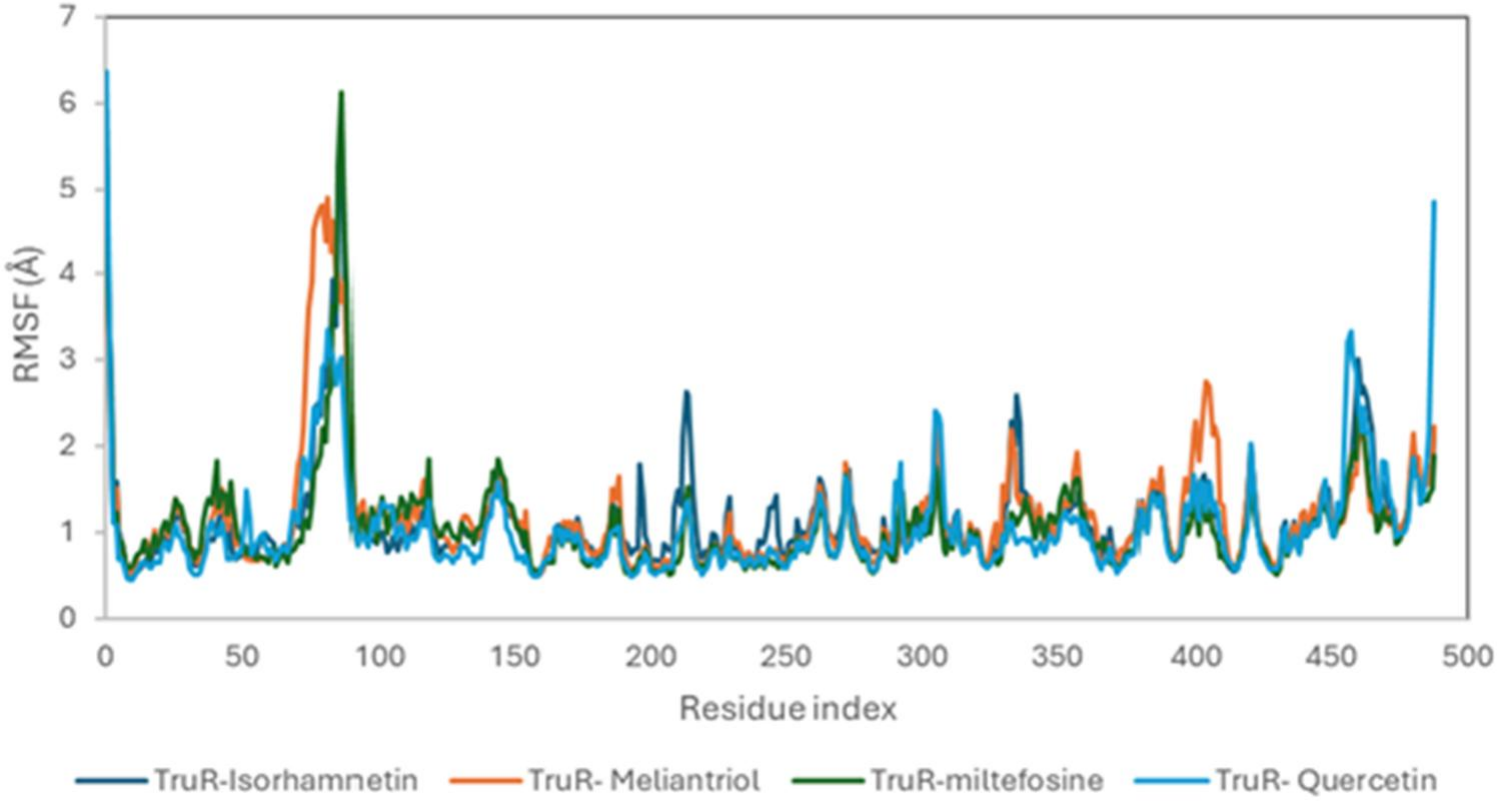
Root mean square fluctuation (RMSF) profiles of TryR in complex with selected ligands during the 100 ns MD simulation. The RMSF (*y*-axis) values represent the flexibility of individual amino acid residues within each complex: TryR–Isorhamnetin, TryR–Meliantriol, TryR–Miltefosine, and TryR–Quercetin. Moderate fluctuations were observed across the protein backbone, with higher peaks indicating regions of greater flexibility, primarily within loop regions, while most residues exhibited low RMSF values.

The root mean square deviation (RMSD), contact plots, and root mean square fluctuation (RMSF) of the complexes during the course of the simulation are presented in Figs 3-5, respectively.

## 4 Discussion

VL is the deadliest of all the clinical manifestations of leishmaniasis; it is the focus of most research efforts to reduce the disease’s global impact. In particular, VL has a high morbidity rate, with previously reported statistics indicating 500 000 new cases and 50 000 fatalities annually. Therefore, by investigating potential inhibitors of TR of *L. infantum*, this study aims to make a substantial contribution to the endeavors aimed at eradicating leishmaniasis caused by *L. infantum*.

Following molecular docking of the compounds to assess their binding affinities to TR, the final docking scores revealed that 10 compounds exhibited high affinity to TR. Notably, rutin exhibited a docking score of −8.482 kcal/mol, while the other compounds also exhibited docking scores lower than the standard drug miltefosine, which had a docking score of −3.231 kcal/mol. Noteworthy, a higher binding affinity is indicated by a lower docking score, suggesting the possibility of forming interactions that could potentially alter the critical structural and functional roles of the target. The top 10 compounds with the highest affinities were selected and subjected to drug-likeness studies tests based on the parameters of the Ro5. Noteworthy, the Ro5 provides simple criteria to determine if a chemical compound has properties consistent with that of orally active drugs (Egbert *et al.* 2019). The four parameters evaluated were: molecular weight (MW) less than 500 Da, hydrogen bond donors (HBD) fewer than 5, hydrogen bond acceptors (HBA) fewer than 10, and predicted octanol/water partition coefficient within −2.0 to 6.5. Compounds violating more than one parameter are considered not drug-like and are excluded from further analysis (Lipinski *et al.* 2012). Of the 10 selected hit compounds, rutin, nicotiflorin, hyperoside, and quercitrin violated the Ro5 parameters beyond the accepted threshold. Specifically, these violations are primarily due to high molecular weight and excessive numbers of hydrogen bond donors and acceptors, suggesting that these compounds are likely unsuitable as orally administered drugs, as they may have poor absorption and low bioavailability. For instance, Rutin and Nicotiflorin show three violations each, suggesting they are less likely to be efficiently absorbed following oral administration. Hyperoside and Quercitrin show two violations, similarly indicating potential limitations in oral delivery. In contrast, the other compounds, including Isorhamnetin, Meliantriol, Quercetin, Nimbinone, Scopoletin, and Methyl-2,5-dihydroxycinnamate, satisfy all Ro5 criteria, suggesting better oral bioavailability potential. Interestingly, these compounds exhibited high affinities for the target. It is important to note that compounds with Ro5 violations may still exhibit biological activity and could serve as lead compounds for chemical modification or alternative administration routes, highlighting their relevance in structure–activity relationship studies (Bourhia *et al.* 2024b).

The ADMET properties of the hit compounds with drug-like characteristics were thoroughly investigated. Human intestinal absorption (HIA), which involves the uptake of drugs from the gastrointestinal system into the systemic circulation after oral administration, is a crucial factor in determining a compound’s efficacy (Aungst 2017). Interestingly, all drug-like hit compounds, including the standard drug miltefosine, were predicted to have high HIA, with scopoletin demonstrating the highest HIA with a value of 95%. While the results of the HIA reveal the compounds as having high absorption potentials, the Caco-2 permeability prediction revealed isorhamnetin, quercetin, and methyl-2,5-dihydroxycinnamate as possessing poor absorption potentials by the Caco-2 cells, which are commonly utilized as an *in vitro* model to predict the absorption of orally administered drugs (Ren and Lien 2000). Conversely, other compounds, including meliantriol, nimbinone, scopoletin, as well as the standard drug (miltefosine) were predicted to have high Caco-2 cell permeability. The contradictory results of the HIA and Caco-2 predictions can be attributed to certain factors, which include the difference in cellular models, as Caco-2 cells are derived from human colon carcinoma and, while widely used as a model for intestinal absorption, they do not perfectly replicate the entire range of conditions and cell types present in the human intestine (Lea *et al.* 2015). Also, a compound might be efficiently absorbed in the human intestine through active transport mechanisms that are not present or not fully functional in Caco-2 cells. Specifically, active transporters, such as the peptide transporters PEPT1 and PEPT2, or other specific transport proteins, can facilitate high absorption in the intestine but are not replicated in the Caco-2 cell line (Lopez-Escalera and Wellejus 2022). Hence, the statistical models used for the analysis differ in their training datasets and consequently give off different results (Pires *et al.* 2015). P-glycoprotein (P-gp), widely distributed throughout the body, is an efflux membrane transporter that plays a crucial role in controlling the cellular absorption and distribution of xenobiotic and harmful chemicals (Amin 2013). Due to its role as an efflux transporter, a drug’s ability to act as a substrate for P-gp can negatively impact its pharmacokinetics and overall effectiveness (Karthika and Sureshkumar 2021). Interestingly, in our study, only the standard drug miltefosine and scopoletin were identified as non-substrates of P-gp, while the other compounds were found to be substrates. This suggests that higher dosages may be necessary to achieve the intended therapeutic efficacy for these P-gp substrates, as their efflux could reduce intracellular drug concentrations. Furthermore, compounds that act as both substrates and inhibitors of P-gp may face restricted applicability in clinical settings. The inhibition of P-gp can lead to significant drug-drug interactions by causing the accumulation of other drugs that rely on P-gp for excretion, potentially resulting in toxicity or reduced efficacy (Bourhia *et al.* 2024b). Therefore, while miltefosine and scopoletin present a pharmacokinetic advantage by not being substrates of P-gp, the substrate status of other compounds warrants careful consideration in dosage planning and potential drug-drug interactions. In contrast to the potential of some of the compounds to serve as substrates of the P-gp, none of the hit compounds were predicted to possess the potential to serve as P-gp I and II, except for the standard drug miltefosine. Regarding metabolism, the inhibition of cytochrome P450 (CYP450) enzymes can significantly impact drug metabolism by reducing the rate of enzyme-catalyzed reactions. This can result in decreased drug metabolism, potentially leading to drug interactions that may impact treatment outcomes and increase the risk of side effects (Deodhar *et al.* 2020). Specifically, isorhamnetin, quercetin, nimbinone, and scopoletin were predicted to inhibit CYP450 1A2, with nimbinone also inhibiting CYP450 2C19. In contrast, meliantriol and miltefosine were predicted to be substrates of CYP450 3A4, indicating that these compounds can undergo phase I metabolism to form appropriate substrates for conjugation reactions facilitated by phase II metabolism enzymes, thereby aiding in their elimination (Zhao *et al.* 2021). Regarding toxicity, all the hit compounds were predicted to be non-mutagenic, as indicated by their negative Ames test results. Additionally, they were found to be non-hepatotoxic, suggesting that their administration would not adversely impact liver function. Furthermore, all the hit compounds were predicted to be non-inhibitors of the hERG 1 channel, which is crucial for cardiac safety, thereby enhancing the overall safety profiles of these compounds.

Molecular Mechanics-Generalized Born Surface Area (MM/GBSA) analysis was performed to assess the binding free energy of the protein-ligand interactions. The hit compounds stably interacted with the amino acid residues of the active site of TR, with meliantriol having the highest binding free energy of −43.09 kcal/mol. MM-GBSA has been reported as a reliable methodology for the validation of docking scores in the absence of experimental data (Lyne *et al.* 2006, Greenidge *et al.* 2013, Tripathi *et al.* 2013). The compounds generally exhibited low Δ*G*_bind_ vdW values, indicating that van der Waals forces significantly contribute to stable binding, and low Δ*G*_bind_ lipophilic values, suggesting that hydrophobic interactions play an important role in stabilizing their complexes with TR (Bourhia *et al.* 2024a).

Interestingly, Miltefosine displayed a positive Δ*G*_bind_ Coulomb and minimal packing energy, in contrast to the other ligands. This can be attributed to its cationic phospholipid structure, which includes a long hydrophobic tail and a charged headgroup. The charged headgroup likely induces electrostatic repulsion with certain residues in the TR active site, while the bulky hydrophobic tail limits optimal steric complementarity, resulting in negligible packing contributions. These features explain why Miltefosine’s Coulomb and packing energies are more significant and less favorable compared to smaller, neutral ligands, highlighting the importance of both electrostatic and steric complementarity in ligand binding.

The interactions of the drug-like hit compounds with TR were examined to identify specific amino acid residues involved in these interactions. Isorhamnetin formed hydrogen bonds with Glu18 and Ser109, hydrophobic interactions with Leu17, Trp21, Met113, and Tyr110, and polar interactions with Ser109 and Ser112 while maintaining a positive interaction with Glu18 and Asp116. Also, meliantriol interacted with TR through hydrogen bonds with Glu18, Asp116, and Tyr110, and hydrophobic interactions with Leu17, Trp21, Val53, Tyr110, Met113, and Leu339. It also formed polar interactions with Thr117, Ser112, Ser14, Ser109, and Thr335, and maintained positive interactions with Glu18 and Asp116. Methyl 2,5-dihydroxycinnamate formed hydrophobic interactions with Trp21, Leu17, Met113, Tyr110, and Ile109, and hydrogen bonds with Ser109 and Tyr110. It also maintained a positive interaction with Glu18 and polar interactions with Ser109. Quercetin formed hydrogen bonds with Glu18 and Ser109, hydrophobic interactions with Met113, Tyr110, Trp21, and Ile330, and polar interactions with Ser112 and Ser109. It maintained a positive interaction with Glu18 and formed a pi-pi interaction with Trp21. Scopoletin interacted with TR through hydrogen bonds with Ser109 and hydrophobic interactions with Trp21, Met113, Tyr110, and Ile106. It also formed polar interactions with Ser112 and Ser109. Nimbinone interacted with TR through hydrogen bonds with Glu49 and Glu13 and hydrophobic interactions with Tyr110, Met113, Leu17, Ile339, Val53, Cys52, Val58, and Cys57. It also formed polar interactions with Ser14 and Thr335 and maintained a positive interaction with Glu18 through hydrogen bonding with Ser14. Conversely, the standard drug miltefosine interacted with TR via hydrogen bonds with Tyr110 and hydrophobic interactions with Leu17, Ile339, Val48, Ile106, Val53, Tyr110, Met113, and Trp21. It also formed polar interactions with Ser14, Thr335, and Ser109, while maintaining a positive interaction with Glu18. Interestingly, Shukla *et al.* similarly reported that the hit compounds of their study exhibited similar interactions with TR, with the ligand forming hydrophobic interactions with Trp21 and Met113 (Shukla *et al.* 2011).

To evaluate the dynamic behavior of the studied complexes in an aqueous medium, the variation of the RMSD was monitored throughout the simulation. Figure 2 shows the plot of RMSD versus time. According to the obtained results, in the presence of miltefosine, the TryR protein underwent conformational changes during the first 60 ns, which are more significant compared to what is observed in the presence of the other ligands. Furthermore, as the simulation progresses, the RMSD values of the Meliantriol ligand become higher than those corresponding to the RMSD of the TryR protein, indicating that the ligand moves away from its initial position at the binding site. Furthermore, the RMSD corresponding to the TryR protein complexed with miltefosine increased during the first 25 ns of the simulation and then relatively stabilized at 2.7 Å, indicating that the protein underwent conformational changes during the initial phase of the simulation to adapt to the aqueous environment in which it is located (Nour *et al.* 2022b, 2023). Furthermore, the RMSD value did not exceed 3 Å, indicating that the protein did not undergo a major conformational change during the simulation (Nour *et al.* 2022a). Almost the same behavior was observed in the presence of Quercetin. In the presence of Isorhamnetin, the RMSD of TryR stabilized after an adaptation period and then increased at the end of the simulation, indicating that the protein is not in equilibrium, and predicts an instability of the TryR-Isorhamnetin complex. Furthermore, the RMSD of Isorhamnetin and miltefosin, increased at the start of the simulation then stabilized at 6 Å, until the end of the simulation. It can be noted that the observed values are not too high compared to the RMSD of the protein, which illustrates that these ligands, compared to Meliantriol, remained relatively close to their initial binding site. However, the RMSD of Quercetin remained relatively constant throughout the simulation, with some negligible fluctuations, illustrating that this ligand remained relatively stable at the binding site. Furthermore, compared to the other ligands, Quercetin remained closest to its initial binding site, reinforcing the stability and coherence of the Quercetin-TryR complex. This observation was confirmed by analyzing the diagrams of the interactions involved between the studied ligands and the target protein during the simulation (Fig. 2). Indeed, according to Fig. 2, Quercetin remained well docked at the TryR binding site by involving permanent interactions with seven amino acids, namely ASP 116, MET 113, TYR 110, SER 109, TRP 21, GLU 18, and SER14, at the binding site. On the other hand, Meliantriol was not able to initiate permanent interactions at the binding site on the target protein, which is explained by the excessively high RMSD values corresponding to this ligand.

The RMSF evolution during the simulation period was also studied and the RMSF diagrams relative to the TryR protein in the presence of the studied ligands are represented in Fig. 3. According to this figure, the four RMSF trajectories have similar shapes, except that there are more intense peaks in some regions of the trajectories corresponding to TryR in the presence of Meliantriol and Quercetin, illustrating the flexibility of its residues (Nour *et al.* 2022c). Additionally, the majority of residues have RMSF values do not exceed 2 Å, reflecting low flexibility of these residues. Furthermore, the residues binding the ligands isomargolonone and Quercetin at the TryR binding site are weakly flexible, which reinforces the stability of the formed complexes.

## 5 Conclusion

Summarily, this aimed to identify probable inhibitors of TryR of *L. infantum* from the phytochemicals of *A. indica* using molecular modeling techniques. To achieve this, the phytochemicals present in the plant were retrieved and subjected to a screening process that assessed their potential as drugs based on the Ro5 and their ADMET properties as well as their binding potentials to TR. The complexes also exhibited stability over a 100 ns simulation. Based on the results of this study, the most promising compounds identified are isorhamnetin, meliantriol, and quercetin. With further experimental validation, these *A. indica* compounds could contribute significantly to the development of treatments for leishmaniasis.

## Supplementary Material

vbaf318_Supplementary_Data

## Data Availability

Data sharing not applicable to this article as no datasets were generated or analyzed during the current study.
