# Supplementary material for: Computational identification of Azadirachta indica compounds targeting trypanothione reductase in Leishmania infantum
Source: Bioinform Adv. 2025 Dec 17;6(1):vbaf318. doi: 10.1093/bioadv/vbaf318 (PMC12776344; doi:10.1093/bioadv/vbaf318)
Supplement: vbaf318_Supplementary_Data [file vbaf318_supplementary_data.docx]

| **Compound** | **SMILES** |
| --- | --- |
| Rutin | CC1C(C(C(C(O1)OCC2C(C(C(C(O2)OC3=C(OC4=CC(=CC(=C4C3=O)O)O)C5=CC(=C(C=C5)O)O)O)O)O)O)O)O |
| Nicotiflorin | CC1C(C(C(C(O1)OCC2C(C(C(C(O2)OC3=C(OC4=CC(=CC(=C4C3=O)O)O)C5=CC(=C(C=C5)O)O)O)O)O)O)O)O |
| Hyperoside | OC([C@H]1O[C@@H](Oc2c(oc3cc(O)cc(O)c3c2=O)-c2ccc(O)c(O)c2)[C@H](O)[C@@H](O)[C@H]1O |
| Quercitrin | Oc1cc(O)c2c(c1)oc(c(c2=O)O)c1ccc(c(c1)O)O (or O=C1c3c(O/C(=C1/O)c2ccc(O)c(O)c2)cc(O)cc3O) |
| Isorhamnetin | COC1=C(O)C=CC(=C1)C1=C(O)C(=O)C2=C(O)C=C(O)C=C2O1 |
| Meliantriol | CC(C)(O)C(O)C1OC(O)CC1C1CCC2(C)C3=CCC4C(C)(C)C(O)CCC4(C)C3CCC12C |
| Quercetin | C1=CC(=C(C=C1C2=C(C(=O)C3=C(C=C(C=C3O2)O)O)O)O)O |
| Nimbinone | C[C@]12CCC(=O)C([C@@H]1CC(=O)C3=CC(=C(C=C23)O)OC)(C)C |
| Scopoletin | [COC1=C(C=C2C(=C1)C=CC(=O)O2)O](https://www.google.com/search?q=COC1%3DC%28C%3DC2C%28%3DC1%29C%3DCC%28%3DO%29O2%29O&sca_esv=b315ce123a2264f5&ei=lc5BaYTOH9_BhbIPgLi28A8&ved=2ahUKEwjc3JHEhsORAxUmU0EAHQPTK3YQgK4QegQIARAE&uact=5&oq=SMILE+of+compound+Scopoletin&gs_lp=Egxnd3Mtd2l6LXNlcnAiHFNNSUxFIG9mIGNvbXBvdW5kIFNjb3BvbGV0aW4yBRAhGKABSJ4MUKgFWKgFcAF4AZABAJgBggSgAYIEqgEDNS0xuAEDyAEA-AEC-AEBmAICoAKUBMICChAAGLADGNYEGEeYAwCIBgGQBgiSBwUxLjQtMaAHsQGyBwM0LTG4B4oEwgcFMC4xLjHIBwmACAA&sclient=gws-wiz-serp&mstk=AUtExfBVkxg2fT98IOB9jWEr5JPiG0TsOwwT248iqtJtAWFALW8eKz4cx13r8LQLuqKVwrNOC9NlaNuPsJkef1mK0K2bkc7L0NobGQsK1kJWyMRUOdDwDzagxHi-jsFJzppwtVyJMEiEealrteJ4t9-T8cTpassv0oEYd3nN2Nobln_3I9flS8CFhw0_JVER35NdtsJn&csui=3) |
| Methyl-2,5-dihydroxycinnamate | COC(=O)/C=C/c1cc(ccc1O)O |
